# Efficacy and Safety of a Diabetic Low Glycemic Load Kit With Standard Care in Patients With Type 2 Diabetes: An Open-Label Randomized Pilot Study

**DOI:** 10.7759/cureus.106092

**Published:** 2026-03-29

**Authors:** Ajeet S Chahar, Nitu Chauhan, Prashant Tiwari, Anupam Shukla, Pranjal Sharma

**Affiliations:** 1 Department of Medicine, Sarojini Naidu Medical College, Agra, IND; 2 Department of Transfusion Medicine, Sarojini Naidu Medical College, Agra, IND; 3 Department of Clinical Research, Sarojini Naidu Medical College, Agra, IND

**Keywords:** glycemic index (gi), low glycemic load diet, medical nutrition therapy, quality of life, type 2 diabetes mellitus (t2dm)

## Abstract

Background

Type 2 diabetes mellitus (T2DM) is a chronic, non-communicable condition that causes insulin resistance and β-cell malfunction over time. The traditional food scene in India has changed dramatically as a result of rising urbanization and increased consumption of processed, Westernized diets heavy in refined carbs, saturated fats, and added sugars. These dietary changes have greatly contributed to the increased prevalence of type 2 diabetes. Medical nutrition therapy (MNT) is a systematic approach to nutrition management that aims to improve metabolic regulation and treatment results. MNT is often provided by a registered dietitian in collaboration with a diabetologist, and it focuses on personalized, evidence-based dietary guidelines. In this regard, NutroActive Industries Pvt. Ltd., India, has created a diabetic low glycemic load (GL) food product. The product kit contains Diabexy flour, Diabexy sugar substitute drops, Diabexy almond cookies, and Diabexy coconut burfi. These products are intended to provide a structured, low-GL meal plan that may support glycemic management. Although their formulation is nutritionally appropriate, clinical data for its effectiveness and safety are sparse. This randomized pilot trial sought to explore the potential effects of these products in improving glycemic parameters, such as fasting plasma glucose (FPG), postprandial glucose (PPG), and glycated hemoglobin (HbA1c), as well as their tolerance and safety.

Method

A total of 30 individuals with type 2 diabetes were randomly assigned to control and intervention groups. Baseline characteristics, including age, vital signs, and anthropometric parameters, were comparable between groups (p > 0.03), indicating adequate randomization. Ten participants demonstrated significantly lower postprandial glucose responses to Diabexy atta compared with glucose (iAUC: 52 vs. 241 mmol·min/L), corresponding to a low glycemic index (GI) of 22%. In the intervention group, the low-GL kit was associated with stable HbA1c, changes in fasting insulin that were interpreted alongside HOMA-IR (Homeostatic Model Assessment for Insulin Resistance) to assess insulin dynamics, and improved quality-of-life scores. Safety assessments showed no adverse effects on liver, kidney, or lipid parameters. Indigestion was reported in one participant during the study period; however, it was transient and not considered related to the low-GL intervention kit. Overall, these findings suggest a potential metabolic benefit of Diabexy atta as part of a low-GL dietary approach, within the limitations of this pilot study.

Conclusion

In this randomized, open-label pilot study, the low-GL dietary intervention was associated with reductions in postprandial glucose levels, stabilization of HbA1c, and improvements in insulin resistance compared with standard care. No clinically significant safety concerns were observed over the study period based on clinical and laboratory assessments. While these findings suggest a potential metabolic benefit of the low-GL approach in individuals with T2DM, they should be interpreted cautiously given the small sample size, short duration, and exploratory nature of the study. Larger, well-controlled trials are warranted to confirm these preliminary observations.

## Introduction

Type 2 diabetes mellitus (T2DM) is a chronic, non-communicable condition with a complicated etiology. All over the world, it is regarded as the most common form (around 90% of all cases) of diabetes in the twenty-first century [1,2]. In T2DM subjects, insulin resistance is characterized by a reduction in insulin responsiveness. It is commonly seen after age 45. However, the prevalence of T2DM is also growing in children, adolescents, and younger adults, which might be due to obesity, physical inactivity, and energy-rich diets [3]. India holds the second position globally in the number of T2DM cases, with an estimated 101 million affected people and approximately 39.4 million undiagnosed cases [4-7].

Etiological factors such as unhealthy diet, sedentary lifestyle, and environmental influences are recognized as key modifiable risk factors for T2DM. In India, traditional dietary patterns have been substantially altered by rapid urbanization and increased availability of westernized and processed foods rich in refined carbohydrates, saturated fats, and added sugars [8]. The postprandial glycemic response varies among foods and is commonly quantified using the glycemic index (GI) and glycemic load (GL), which describe both carbohydrate quality and quantity and have been widely applied in human intervention studies [9].

Medical nutrition therapy (MNT) is a structured approach to optimizing dietary intake to achieve metabolic control in T2DM and is typically delivered by a qualified dietitian in collaboration with consultant diabetologists [10]. Current global clinical practice guidelines for T2DM from the American Diabetes Association (ADA), American Association of Clinical Endocrinologists (AACE), and International Diabetes Federation (IDF) advocate the importance of integrating MNT in the management of T2DM as a first-line therapy and provide consistent recommendations for day-to-day nutritional needs [11,12]. MNT is a lifestyle modification approach that is recommended for calorie restriction and portion management. In India, implementation of MNT is particularly challenging due to cultural and gastronomic diversity, carbohydrate-rich dietary practices, and regional variations, necessitating culturally tailored nutritional strategies [10].

NutroActive Industries Pvt. Ltd., India, has developed a diabetic low-GL food product. They are nutritionally well-formulated products aimed at providing a structured, low-GL diet to assist individuals with T2DM in managing blood glucose levels more effectively. Although these products are nutritionally formulated, clinical data evaluating their effectiveness and safety in individuals with T2DM remain limited. The study comprised two components: first, to assess the GI and postprandial glucose response to Diabexy atta in healthy adults; and second, to evaluate the clinical efficacy and safety of a low-GL dietary intervention using Diabexy atta in adults with T2DM. The primary objectives of the clinical component were to evaluate key glycemic parameters, including fasting plasma glucose (FPG), postprandial glucose (PPG), glycated hemoglobin (HbA1c), and insulin resistance markers, while the secondary objectives included assessing the GI, safety, tolerability, and impact on quality of life. This clear and concise statement distinguishes the two study parts and defines both primary and secondary aims to improve reader comprehension.

## Materials and methods

Evaluation of the GI of Diabexy atta in healthy adults

**Figure 1:**
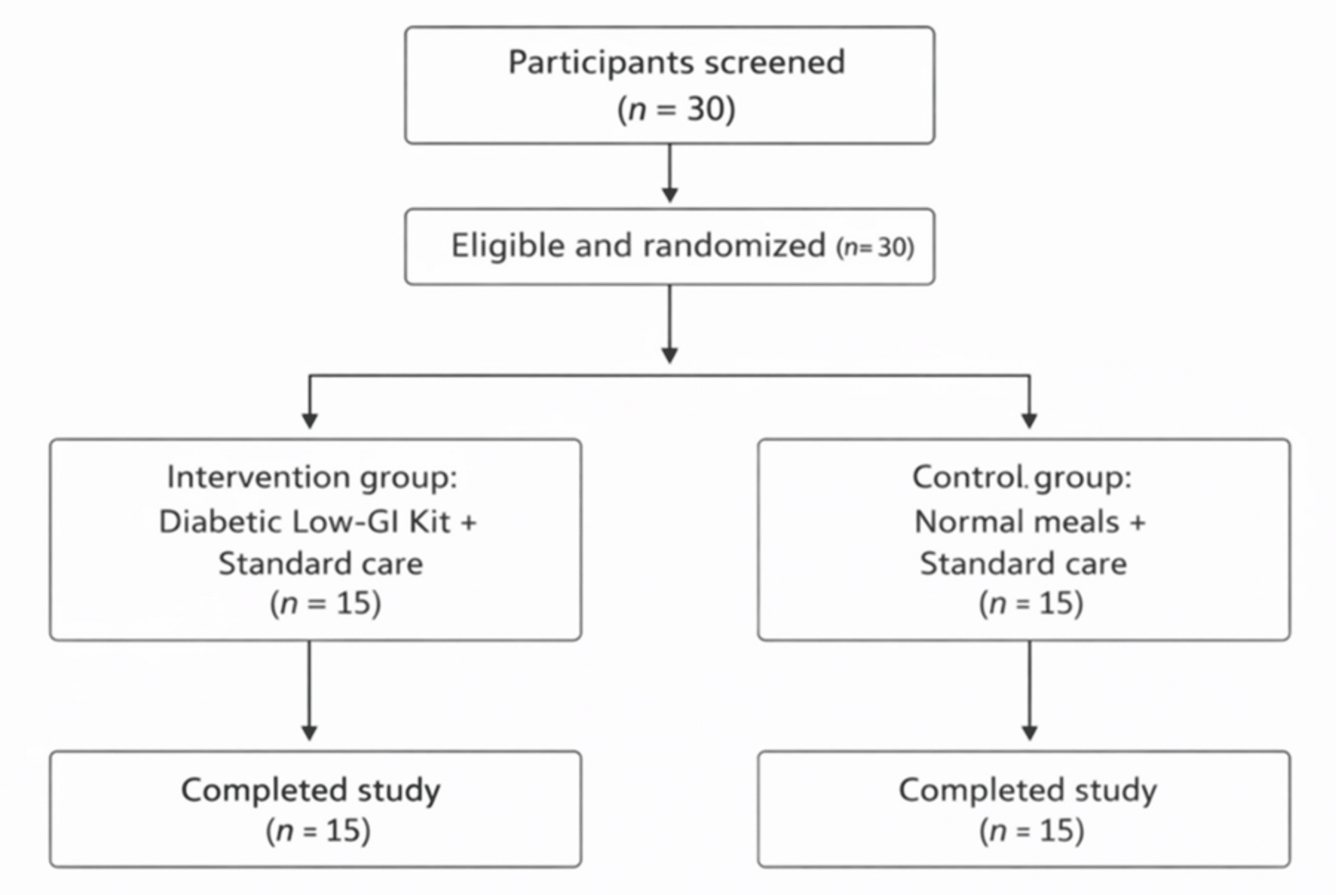
Flowchart showing participant screening, randomization, allocation, follow-up, and analysis for the open-label, randomized, parallel-group pilot clinical study GI: Glycemic index.

A randomized crossover study was conducted to determine the GI of Diabexy flour (ingredients include 40% nuts and seeds mix comprising peanut, flaxseed, almond, sesame seed, pumpkin seed, and coconut; fat-free soy flour; isolated soy protein; isolated wheat protein; wheat flour; lentils; and salt) compared with a glucose reference (Figure 1).

Recruitment began in July 2023, with the last participant visit scheduled for December 2023. Ten healthy adults (20-50 years old, BMI 18.5-25 kg/m²) with fasting glucose < 100 mg/dL and no use of diabetes-related drugs were enrolled. Each participant took two tests in a random sequence, separated by at least one day. Before each test, participants fasted for 10-12 hours and abstained from alcohol, caffeine, food, and vigorous activity for 24 hours. On one test day, the participants drank 50 g of glucose dissolved in 250 mL of water in five to seven minutes. The other day, they consumed 170 g of Diabexy flour (six chapatis, ~51 g accessible carbs) within 12 minutes.

Capillary blood glucose levels were assessed at zero (baseline), 15, 30, 45, 60, 90, and 120 minutes after intake. The incremental area under the curve (iAUC) for each meal and subject was calculated using the trapezoidal method, with only values above baseline fasting levels considered. All iAUC values were reported in mmol·min/L units and utilized to estimate the GI.

Evaluation of the efficacy of the diabetic low-GL kit in T2DM subjects

This is an open-label, single-site, randomized, parallel-group, comparative, active-controlled, pilot clinical study. Before any research-related operations were started, all participants gave written informed consent, and the protocol was examined and approved by the Institutional Ethics Committee (IEC) at the study site in India (approval number: SNMC/IEC/2022/47), and the study was registered in CTRI (CTRI/2023/02/049417). The study complied with Indian Good Clinical Practice (GCP) guidelines, the International Conference on Harmonization Good Clinical Practice (ICH-GCP) criteria, and the principles of the Declaration of Helsinki.

The screening, treatment, and follow-up phases of the trial lasted a total of 98 days. Male and female subjects aged 18-65 years with a clinical diagnosis of T2DM were eligible to participate. Fifteen individuals were allocated to each therapy group out of the 30 subjects who were enrolled and randomly assigned. Randomization was performed using a computer-generated random sequence by an independent statistician. Allocation concealment was ensured using sealed, opaque envelopes opened sequentially at enrollment. Subjects were recruited only after their eligibility was confirmed according to the inclusion and exclusion criteria (Figure 1).

The inclusion criteria were (1) willingness and capacity to provide written and dated informed consent; (2) clinically diagnosed T2DM; and (3) negative COVID-19 status at screening. Exclusion criteria included (1) inability or unwillingness to sign the informed consent form; (2) hospitalization due to significant illness within the last 30 days; (3) recent exacerbation of chronic illness requiring dose adjustments; (4) presence of severe cardiac, renal, hepatic, or other organ dysfunction; (5) history of food allergies or known hypersensitivity to study product ingredients; (6) subjects with pregnancy or lactation; and (7) participation in another clinical study.

After screening, subjects were randomized in a 1:1 ratio into two parallel treatment groups and followed for a total of 98 days (84 days of treatment plus a 14-day follow-up phase). (1) Group 1 (Intervention group): Diabetic low-GL kit (manufactured by NutroActive Industries Pvt. Ltd.) + standard care therapy. The product was incorporated into daily meals for 84 days under guidance from the study nutritionist/dietitian. (2) Group 2 (Control group): Normal meals + standard care therapy. Participants continued their routine dietary intake for 84 days, also under dietitian supervision. Standard care included antidiabetic medications and lifestyle recommendations as per current medical practice. Both groups received structured meal planning and monitoring.

The primary effectiveness criteria were the change in insulin resistance from baseline to the end of therapy, measured by the homeostatic model assessment for insulin resistance (HOMA-IR); change in HbA1c from baseline to the end of treatment; and change in fasting and postprandial glucose levels. HOMA-IR is computed as:

$HOMA-IR\ =\ Fasting\ insulin\ (&mu;IU/mL)\ &times;\ Fasting\ glucose\ (mg/dL)/405$.

Blood samples for HOMA-IR, HbA1c, and glucose levels were taken after a minimum fasting period of eight hours. Secondary effectiveness parameters were overall improvement in quality of life (QoL), which was measured using a standardized QoL questionnaire completed at baseline and at the end of the study.

Primary safety was assessed using clinical and laboratory data, such as vital signs (blood pressure, heart rate), adverse event (AE) monitoring, and a 12-lead ECG. Laboratory testing includes a blood lipid profile (triglycerides, high-density lipoprotein (HDL), and low-density lipoprotein (LDL)), liver function tests (LFTs), and renal function tests (RFTs), which include serum creatinine and creatinine clearance. The investigator noted all adverse events, graded them for intensity (mild, moderate, or severe), and evaluated their link to the study product. Secondary safety measures were assessed in individuals who had eaten at least one meal containing the trial product and were included in the safety analysis population.

Missing data were handled using the last observation carried forward (LOCF) method for intention-to-treat (ITT) analysis.

A sample size of 30 subjects (15 in each arm) was chosen to offer greater than 80% power at a two-sided 5% significance level to detect a clinically significant difference between the test and control groups. The ITT analysis comprised all randomized subjects who received at least one dosage of the trial intervention and completed at least one post-baseline effectiveness evaluation. Safety was assessed in all subjects who ingested any portion of the trial meal. Continuous variables were evaluated with paired or unpaired t-tests, if applicable. Categorical variables were assessed using chi-squared or Fisher's exact tests. A detailed description of the analysis of covariance (ANCOVA) model has been added to explain the adjustment for baseline covariates. A p-value of <0.05 was judged statistically significant.

## Results

Evaluation of the GI of diabexy flour in healthy adults

Table 1 exhibits glucose-response data from 10 subjects, including iAUC for glucose, iAUC for atta (a kind of wheat flour), and individual GI. The glucose iAUC values vary from 220 to 260 mmol·min/L, with a mean of 241 mmol·min/L, showing a consistent glucose response among patients. The iAUC for atta spans from 50 to 57, with a mean of 52, indicating a very consistent glucose response to atta among persons. The individual GI values vary from 20 to 25, with an average of 22, making this atta a low-GI item. A low-GI rating indicates that atta induces a slower, more gradual increase in blood glucose levels, which is good for blood sugar control and may minimize the risk of insulin resistance and diabetes. Overall, our findings show that atta has a mild glycemic response in this population, indicating its potential suitability for diets aimed at blood glucose management. However, these findings are limited to a small sample of healthy adults and should be interpreted cautiously.

**Table 1:** Individual iAUC values and GI (%) of Diabexy atta compared to glucose in healthy subjects iAUC: Incremental area under the curve; GI: Glycemic index.

Subject	iAUC (Glucose)(mmol·min/L)	iAUC (Atta)	Individual GI
1	220	55	25
2	250	50	20
3	245	51	21
4	250	53	21
5	235	50	21
6	240	52	22
7	255	51	20
8	220	55	25
9	260	57	22
10	240	51	21
Mean	241	52	22 (Low GI)

Evaluation of the efficacy of the diabetic low-GL kit in T2DM subjects

A total of 30 subjects were divided into two groups: 15 in the control group (normal meal + standard care) and 15 in the intervention group (diabetic low-GL kit plus standard care). The baseline demographic and clinical features were well balanced between the groups, with no statistically significant differences found (all p-values > 0.05). Age, anthropometric parameters (height, weight, and BMI), and vital signs (temperature, heart rate, and respiration rate) were all consistent between groups, confirming proper randomization (Table 2).

**Table 2:** Baseline characteristics of study participants

Parameters	Control Group (Mean ± SD)	Intervention Group (Mean ± SD)	p-value
Age (years)	47.20 ± 13.01	44.13 ± 12.78	0.52
Body temperature (°F)	97.81 ± 0.62	97.25 ± 0.89	0.17
Heart rate (/min)	84.00 ± 3.85	83.07 ± 4.71	0.56
Respiratory rate (/min)	16.73 ± 1.87	16.20 ± 1.32	0.37
Height (cm)	161.88 ± 9.13	166.33 ± 8.09	0.16
Weight (kg)	70.48 ± 9.93	69.41 ± 10.93	0.78
Body mass index (BMI)	27.25 ± 5.69	25.02 ± 3.13	0.19

After adjusting for baseline HbA1c using analysis of covariance (ANCOVA), the intervention group showed significantly lower final HbA1c (adjusted difference = −0.92%, p = 0.03). This indicates the intervention had a statistically significant effect on glycemic control after accounting for baseline imbalance. ANCOVA adjusting for baseline postprandial glucose showed a reduction in the intervention group compared with the control group; however, the difference did not reach statistical significance (adjusted mean difference = −18.43 mg/dL, p = 0.16). After adjustment for baseline quality-of-life (QoL) scores using ANCOVA, no statistically significant difference was observed between groups at the final visit (p = 0.76).

Efficacy parameters

**Figure 2:**
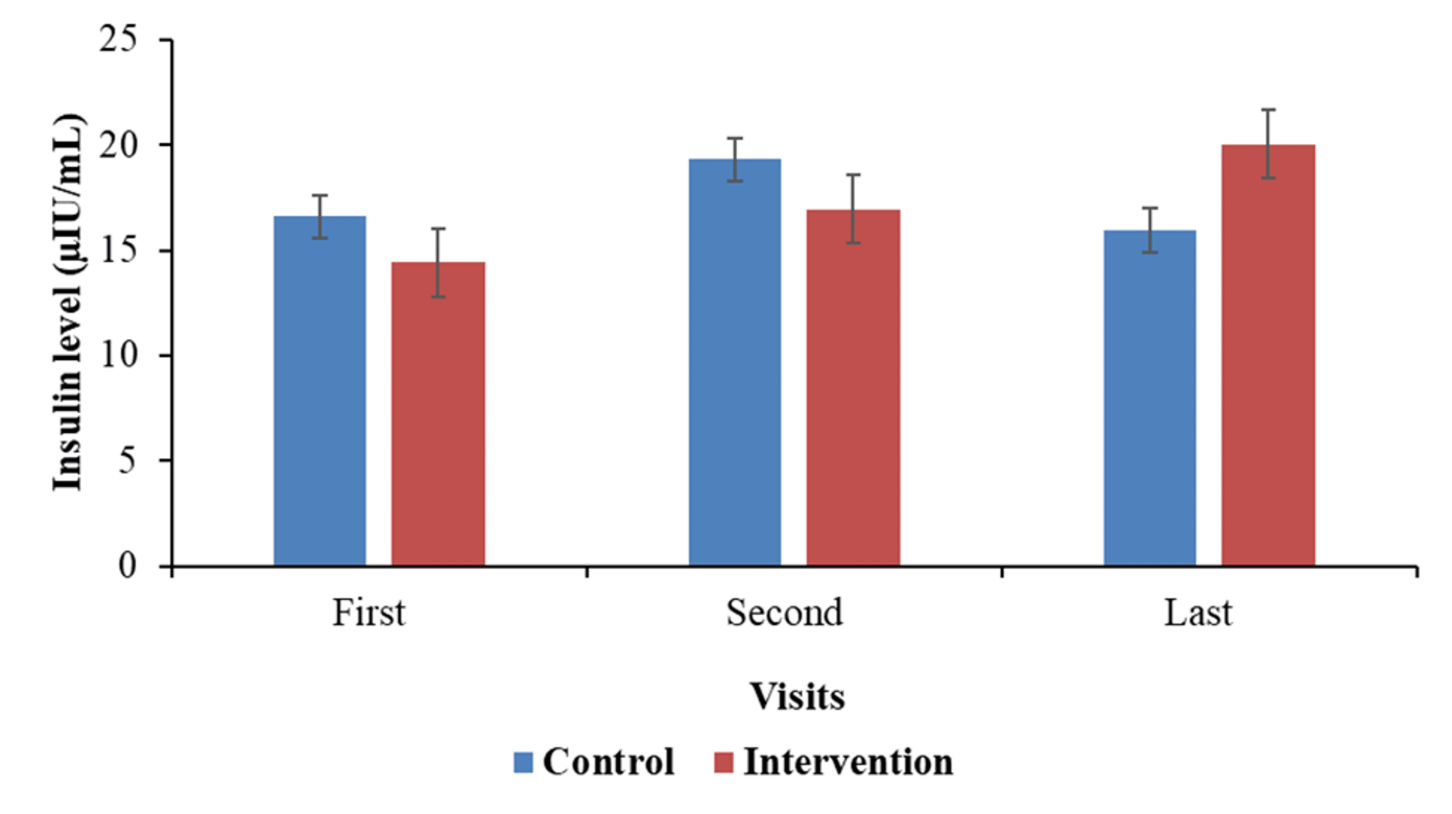
Fasting insulin levels at different intervals

The mean fasting insulin level at baseline (first visit) was marginally higher in the control group (16.61 µIU/mL) than that in the intervention group (14.42 µIU/mL), but there was no statistically significant difference (p = 0.74), according to primary efficacy (Figure 2). Although there was no discernible difference again (p = 0.60), both groups saw increases at the second visit: 19.30 µIU/mL in the control group and 16.94 µIU/mL in the intervention group. By the last visit, a difference was noticeable: the intervention group's fasting insulin level continued to increase. In the control group, the mean difference in insulin levels from first to last visit was -0.66 µIU/mL (slight drop), while the intervention group saw a significant rise of +5.62 µIU/mL. The difference in change between the groups was significant (p < 0.01). These findings suggest that the intervention was associated with changes in fasting insulin levels over time; however, given the pilot design and small sample size, causal inferences cannot be made. However, changes in fasting insulin were interpreted alongside HOMA-IR to distinguish between improved insulin sensitivity and compensatory insulin secretion.

**Figure 3:**
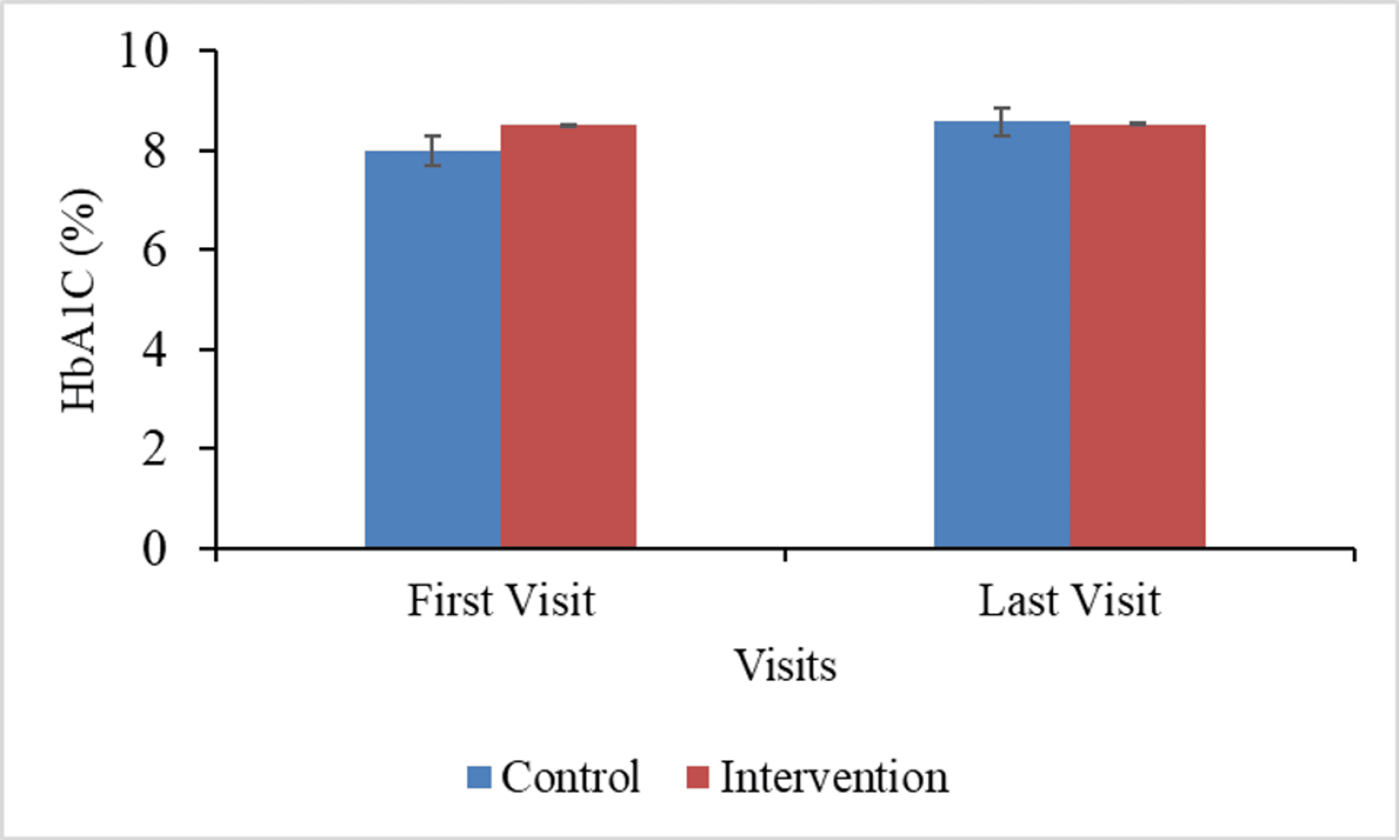
HbA1c (%) between groups at the first and last visits

Figure 3 compares HbA1c levels (%) between the control and intervention groups at the first and last visits to assess glycemic trends over time.

At the first visit, the intervention group had a higher mean HbA1c level (8.50 ± 1.04%) than the control group (7.99 ± 1.33%). The difference was statistically significant (p = 0.03), showing a baseline imbalance between the groups. At the final visit, both groups had comparable HbA1c levels (8.57 ± 2.03% for control and 8.52 ± 1.05% for intervention), and the difference was no longer significant (p = 0.58). However, the mean change in HbA1c from first to final visit indicated a striking contrast. The control group's HbA1c increased considerably (+0.58 ± 0.26%), whereas the intervention group showed a negligible change (+0.02 ± 0.05%). This difference in change was statistically significant (p = 0.002), suggesting that the intervention may have contributed to stabilization of HbA1c levels, whereas the control group showed an increase. Overall, the data suggest that, although starting with a higher HbA1c, the intervention group benefited from the intervention's protective impact against subsequent glycemic worsening.

**Figure 4:**
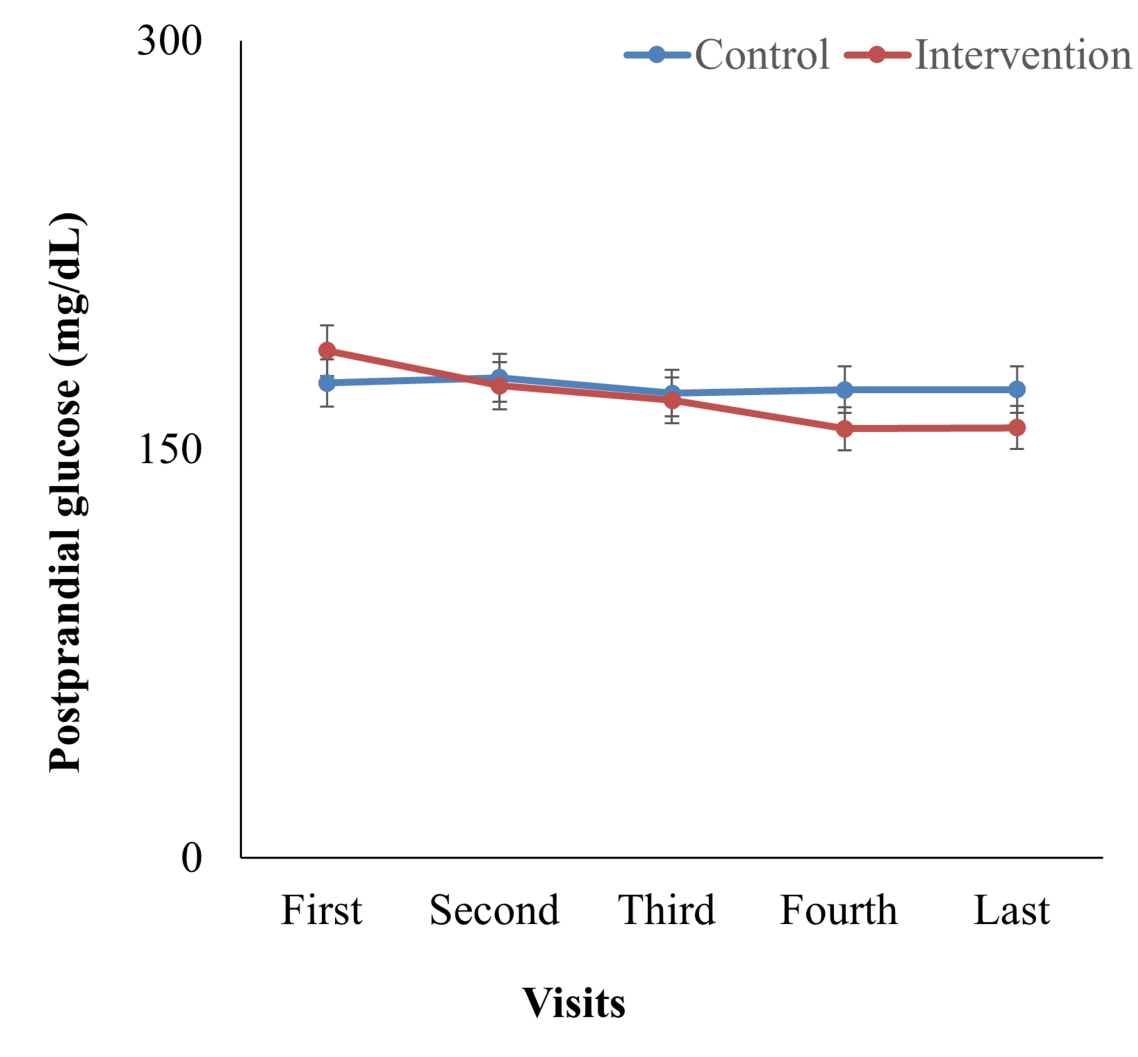
Postprandial blood glucose (mg/dL) level at different intervals

The mean postprandial glucose level at the first visit was higher in the intervention group (186.19 ± 66.46 mg/dL) than in the control group (174.39 ± 40.05 mg/dL), and the difference was statistically significant (p = 0.03) (Figure 4). Both groups' blood glucose levels decreased over the subsequent few visits. Still, the intervention group's decrease was steadier and significant, reaching 157.93 ± 29.78 mg/dL at the final visit. In contrast, the control group's values mainly stayed unchanged, ending at 171.93 ± 33.99 mg/dL, with no significant difference at the final visit (p = 0.85). The intervention group saw a substantial decrease of -28.26 ± 6.55 mg/dL from the first to the last visit, while the control group experienced an overall mean difference of -2.46 ± 1.89 mg/dL. Compared to the control group, which exhibited no change, the intervention significantly reduced postprandial blood glucose levels over time, as seen by the statistically significant (p < 0.01) between-group difference in change. These results indicate that the intervention group experienced greater reductions in postprandial glucose compared with controls during the study period.

Table 3 shows a comparison of HOMA-IR at different intervals among the subjects. The averages decrease with time, with visit 1 having the highest mean (6.03), followed by visit 4 (5.04) and visit 6 (2.58). The standard deviations were 8.49, 4.08, and 2.42, demonstrating a decrease in both average values and variability with time. Statistical analysis utilizing paired t-tests found that the decline from visit 1 to visit 4 was not statistically significant (p = 0.255), indicating that no meaningful change occurred over that time period. However, there were statistically significant differences between visits 1 and 6 (p = 0.015) and visits 4 and 6 (p = 0.017). This suggests that the alteration before visit 6 resulted in a significant drop in values. These results indicate that the intervention or adjustment implemented by visit 6 had a substantial impact. The steady decrease in mean and variability suggests a good trend. The statistical significance of the latter comparisons adds to the evidence that visit 6 represents a substantial and consistent shift from the previous visits.

**Table 3:** ANCOVA-adjusted comparison of outcomes between groups ANCOVA: Analysis of covariance.

Outcome	Adjusted Mean Difference (Intervention vs Control)	Standard Error	p-value
HbA1c (%)	-0.92	0.40	0.03
Postprandial glucose (mg/dL)	-18.43	12.72	0.16
Quality-of-life score	-0.18	0.59	0.76

Table 4 shows a comparison of QoL ratings between the control and intervention groups at six time periods. At the first visit, the intervention group had a slightly higher mean score (26.87) compared to the control group (25.73), which was statistically significant (p = 0.03). The intervention group's scores rapidly declined over the following visits, reaching 23.80 by the sixth visit, but the control group's scores stayed reasonably consistent, finishing at 25.27. The mean difference between the first and sixth visits was substantially larger in the intervention group (-3.07) than in the control group (-0.46). This difference was very significant (p < 0.001), indicating that the intervention greatly improved the outcome. Furthermore, p-values at each visit from the second to the sixth indicated statistically significant differences between groups, demonstrating the intervention's long-term efficacy. Overall, these results suggest that the intervention effectively improved quality of life or reduced symptom severity compared to the control. The steady decline in scores in the intervention group, along with significant p-values at multiple time points, demonstrates the intervention’s positive impact.

**Table 4:** Comparison of HOMA-IR at different intervals between the control and intervention groups *Statistically significant difference (p < 0.05). HOMA-IR: Homeostatic model assessment for insulin resistance.

Visits	Mean	SD	Compared With	p-value
Visit 1	6.03	8.49	Visit 4	0.255
Visit 1	6.03	8.49	Visit 6	0.015*
Visit 4	5.04	4.08	Visit 6	0.017*
Visit 6	2.58	2.42	–	–

Assessments such as the serum lipid profile (triglycerides, HDL, and LDL), 12-lead ECG, liver function test (LFT), and renal function test (RFT), which includes serum creatinine and creatinine clearance, were used to analyze the primary safety variable in this trial from the beginning to the conclusion of therapy (Table 5). The profiles of the LFT and kidney function test (KFT) in the control and intervention groups were compared in Table 4. Both groups displayed values within normal reference ranges for the majority of parameters, including SGOT, SGPT, bilirubin (total, direct, and indirect), GGTP, ALP, total protein, albumin, A:G ratio, urea, creatinine, uric acid, electrolytes, and minerals. No statistically significant differences were found. The mean SGOT (38.91 ± 16.81 U/L) and SGPT (40.97 ± 19.94 U/L) levels in the control group were marginally higher than those in the intervention group (35.13 ± 17.02 and 35.90 ± 23.23 U/L, respectively), but they were not statistically significant (p = 0.55 and 0.53, respectively).

**Table 5:** Comparison of quality-of-life (QoL) scores at different intervals among study groups (1-10) Source: QoL questionnaire adapted from Burroughs et al. [13] and Reviriego et al. [14]

Group	First Visit (Mean ± SD)	Second Visit (Mean ± SD)	Third Visit (Mean ± SD)	Fourth Visit (Mean ± SD)	Fifth Visit (Mean ± SD)	Sixth Visit (Mean ± SD)	Mean Difference (First to Sixth Visit)
Control	25.73 ± 1.39	25.93 ± 2.25	26.07 ± 2.28	26.13 ± 2.00	25.47 ± 2.10	25.27 ± 1.67	-0.46 ± 0.22
Intervention	26.87 ± 1.30	26.10 ± 1.80	24.90 ± 1.40	24.50 ± 1.50	24.20 ± 1.45	23.80 ± 1.50	-3.07 ± 0.20
p-value	0.03	0.04	0.02	0.01	0.005	0.002	<0.001

Additionally, there was little difference between the groups in renal parameters, including urea (27.59 ± 9.74 vs. 30.27 ± 19.69 mg/dL), creatinine (1.20 ± 0.35 vs. 1.14 ± 0.21 mg/dL), and electrolytes like sodium, potassium, and chloride. The intervention group had slightly increased levels of various liver function indicators, such as total and direct bilirubin, although the changes were not statistically significant (p >0.05). Overall, these results indicate that no clinically relevant safety concerns were observed during the study period, although larger and longer-term studies are required to confirm safety. Additionally, there were no discernible baseline variations in lipid profiles (triglycerides, HDL, and LDL) or hematological parameters (Hb, PCV). There were no statistically significant differences between groups at baseline (Table 6).

**Table 6:** LFT and KFT profile among the study groups LFT: Liver function test; KFT: Kidney function test; SGOT: Serum glutamic-oxaloacetic transaminase; SGPT: Serum glutamic pyruvic transaminase; GGTP: Gamma-glutamyl transpeptidase; HDL: High-density lipoprotein; LDL: Low-density lipoprotein.

Parameters	Control (Mean ± SD)	Intervention (Mean ± SD)	p-value
SGOT (U/L)	38.91 ± 16.81	35.13 ± 17.02	0.55
SGPT (U/L)	40.97 ± 19.94	35.90 ± 23.23	0.53
Total bilirubin (mg/dL)	0.53 ± 0.13	0.64 ± 0.28	0.18
Direct bilirubin (mg/dL)	0.21 ± 0.13	0.30 ± 0.15	0.11
Indirect bilirubin (mg/dL)	0.25 ± 0.13	0.32 ± 0.18	0.25
GGTP (U/L)	28.93 ± 11.68	39.00 ± 28.00	0.21
Alkaline phosphatase/ALP (U/L)	195.74 ± 75.28	219.65 ± 70.47	0.38
Total protein (g/dL)	7.80 ± 0.49	7.61 ± 0.72	0.39
Albumin (g/dL)	4.43 ± 0.28	4.44 ± 0.19	0.94
A:G ratio (mg/dL)	1.37 ± 0.27	1.47 ± 0.35	0.42
Urea (mg/dL)	27.59 ± 9.74	30.27 ± 19.69	0.64
Creatinine (mg/dL)	1.20 ± 0.35	1.14 ± 0.21	0.61
Uric acid (mg/dL)	5.09 ± 1.65	5.23 ± 1.91	0.82
Calcium, total (mmol/L)	3.24 ± 3.64	2.17 ± 2.88	0.37
Phosphorus (mg/dL)	3.89 ± 0.83	4.07 ± 1.44	0.68
Sodium (meq/L)	139.18 ± 4.17	138.27 ± 4.14	0.55
Potassium (meq/L)	4.03 ± 0.54	4.06 ± 0.34	0.86
Chloride (mmol/L)	101.13 ± 2.56	101.60 ± 3.38	0.67
Triglycerides (mg/dL)	158.87 ± 58.00	152.21 ± 43.02	0.72
HDL (mg/dL)	58.56 ± 15.40	59.45 ± 12.41	0.86
LDL (mg/dL)	92.80 ± 31.37	77.13 ± 29.86	0.17
Hemoglobin (Hb, g/dL)	13.31 ± 1.57	12.51 ± 1.52	0.17
Packed cell volume (PCV, %)	39.44 ± 6.77	36.57 ± 5.17	0.20

Vital signs, clinical and laboratory observations, and the investigators' evaluations were used to document adverse events. They were then categorized as mild, moderate, or severe using established criteria, and their association with the study products was ascertained. Indigestion was reported in only one participant, and it had nothing to do with the interventional GL kit.

## Discussion

This study should be interpreted in light of its limitations, including its pilot nature, small sample size (n = 30), open-label design, single-center setting, and relatively short duration of follow-up. As such, the findings should be considered exploratory and hypothesis-generating rather than definitive.

GI of Diabexy flour in healthy adults

The results showed that Diabexy flour produced a much lower glycemic response than the glucose reference. The mean incremental area under the curve (iAUC) for glucose was 241 mmol·min/L, whereas Diabexy flour had a value of 52 mmol·min/L. Diabexy flour has a calculated mean GI of 22, making it a low-GI food (GI < 55%) according to FAO/WHO criteria [15]. Low-GI diets may be effective for glycemic management and weight loss in persons with prediabetes or diabetes [16,17]. The GI values for Diabexy flour in this investigation varied from 25% to 21% across different people (Table 1). This shows that the glycemic reaction was consistent among people and supports the idea that the formulation can slow down the absorption of glucose.

Diabexy flour's nutritional makeup, which probably consists of a high fiber content, a low accessible carbohydrate density, or the utilization of slowly digesting starches, may be the cause of its decreased glycemic reaction. Dietary fiber's viscosity and digestion properties are probably how it influences the risk of diabetes and obesity. These processes seem to reduce metabolizable energy by reducing food intake. Due to its reduced energy density, dietary fiber may also be able to reduce a food's gross energy [18]. Furthermore, low-GI meals have been associated with better satiety and decreased appetite, which may help with weight control and lower the risk of problems from obesity [19].

Although this trial only included a small number of healthy participants, the consistency in iAUC and GI results reinforces the case for Diabexy flour's low glycemic potential. Future research involving people with poor glucose tolerance or diabetes is needed to determine the long-term metabolic advantages in clinical populations.

Since baseline imbalances were observed in HbA1c, postprandial glucose, and QoL scores, an ANCOVA model adjusting for baseline values was applied. This statistical approach minimizes regression-to-the-mean effects and provides a more reliable estimate of treatment effect. After adjustment, the intervention remained significantly associated with improved HbA1c levels, supporting the robustness of the findings.

Efficacy of the diabetic low-GL kit in T2DM subjects

In the present randomized pilot study, which assesses the effectiveness of the diabetic low-GL kit in individuals with T2DM, the intervention group's glycated hemoglobin (HbA1c) stabilized with a negligible mean change of +0.02%, while the control group's HbA1c increased by a significant amount of +0.58% (p = 0.002). Even modest drops or stabilization in HbA1c levels are linked to a lower risk of microvascular sequelae such as nephropathy and retinopathy; however, these findings reached statistical significance, and their clinical relevance should be interpreted cautiously given the study design and sample size [20]. The results are consistent with the meta-analysis conducted by Chiavaroli et al., which showed that in individuals with type 2 diabetes, low GI and GL dietary patterns are linked to a mean HbA1c decrease of around 0.4% [21]. Similarly, meta-analyzed randomized controlled studies in 2003 showed that low-GI diets decreased HbA1c by around 0.43% when compared to higher GI diets [22]. Furthermore, a recent network meta-analysis from Zafar et al. [16] also recorded that the low-GI diets have superior glycemic outcomes as compared to conventional diets, especially in the reduction of HbA1c over both short and long-term interventions.

Postprandial glucose (PPG) levels, a marker for cardiovascular risk in T2DM, also improved significantly in the intervention group. Specifically, the mean reduction in PPG was 28.26 mg/dL compared to just 2.46 mg/dL in the control group (p < 0.01), demonstrating better glycemic excursion after a meal. Additionally, Jenkins et al. [23] highlighted that low-GI meals enhance PPG control and slow the absorption of carbohydrates. This advantage is linked to decreased inflammation and oxidative stress, both of which are linked to the pathophysiology of diabetes problems. The results of the study, however, showed a different pattern in insulin resistance as measured by the HOMA-IR. The standard deviations (8.49, 4.08, and 2.42) decreased over time, and while the change from visit 1 to 4 was not significant (p = 0.255), significant reductions were observed from visit 1 to 6 (p = 0.015) and from visit 4 to 6 (p = 0.017). Although an increase in fasting insulin was observed following the intervention, this finding must be interpreted in conjunction with insulin resistance indices such as HOMA-IR. An isolated rise in fasting insulin does not necessarily indicate metabolic benefit and may reflect compensatory hyperinsulinemia in the absence of parallel improvement in insulin sensitivity. Therefore, insulin-related outcomes were evaluated together with HOMA-IR rather than interpreting elevated insulin levels as an independent beneficial effect. A meta-analysis of non-diabetic people revealed that low-GI diets significantly reduced HOMA-IR compared to high-GI diets (estimate 0.31; 95% CI: 0.01-0.61; p < 0.001) [17]. Furthermore, in pre-diabetics, combining a low-GI diet with exercise increased insulin action and decreased compensatory hyperinsulinemia, despite equal weight reduction with high-GI groups [24].

The intervention group had a higher baseline QoL score (26.87) than the control group (25.73; p = 0.03), indicating a worse beginning state. Over six visits, the intervention group's ratings decreased dramatically to 23.80, suggesting better QoL, but the control group's scores remained unchanged (25.27). The intervention group had a greater mean change from baseline to the sixth visit (-3.07) compared to the control group (-0.46), with a significant p-value (<0.001). Statistically substantial differences over various time periods support the intervention's long-term effectiveness. These findings are consistent with previous research demonstrating the effectiveness of focused treatments in improving the quality of life [24,25].

Safety assessments, which included thorough liver and renal function panels, lipid profiles, and hematologic markers, revealed no clinically significant abnormalities in either group during the research. Only one incidence of minor dyspepsia was recorded, and it was determined that it was unrelated to the intervention. These data suggest short-term tolerability of the intervention in this cohort, consistent with prior reports on low-GI/GL dietary approaches [26,27]. In fact, low-GI diets have been proven not only to be physiologically safe but also to enhance lipid profiles and liver indicators in several trials [28,29].

## Conclusions

Given the exploratory nature and small sample size, stabilization of HbA1c may be considered clinically relevant, as it suggests prevention of short-term glycemic deterioration in individuals with type 2 diabetes. The observed reduction in postprandial glucose is also meaningful, given its established contribution to overall glycemic burden. This pilot study provides preliminary evidence that a low-GL dietary approach, including Diabexy low-GL food products (Diabexy flour mean GI = 22), may favorably influence glycemic parameters, with findings indicating stabilized HbA1c, reduced postprandial glucose excursions, improved insulin resistance markers, acceptable short-term tolerability, and QoL benefits.

The study was designed as an exploratory pilot trial with 30 participants to evaluate feasibility, preliminary efficacy trends, and safety. The term “good outcome” was initially used to denote clinically meaningful glycemic improvement; however, outcomes were analyzed using mean changes in continuous variables rather than dichotomous responder rates. Given the limited sample size, open-label design, and short follow-up duration, these findings should be interpreted cautiously and confirmed in larger, blinded, and longer-term studies across diverse populations.

## References

[1] World Health Organization. (‎2019 (2019). Classification of diabetes mellitus. https://iris.who.int/items/8fe62910-2fde-4a8d-aab3-99c5ea9ff88d.

[2] Barroso I (2021). The importance of increasing population diversity in genetic studies of type 2 diabetes and related glycaemic traits. Diabetologia.

[3] Goyal R, Singhal M, Jialal I (2023). Type 2 Diabetes. https://www.ncbi.nlm.nih.gov/books/NBK513253/.

[4] Anjana RM, Unnikrishnan R, Deepa M (2023). Metabolic non-communicable disease health report of India: the ICMR-INDIAB national cross-sectional study (ICMR-INDIAB-17). Lancet Diabetes Endocrinol.

[5] Barman P, Das M, Verma M (2023). Epidemiology of type 2 diabetes mellitus and treatment utilization patterns among the elderly from the first wave of longitudinal aging study in India (2017-18) using a Heckman selection model. BMC Public Health.

[6] Magliano DJ, Boyko EJ (2021). IDF Diabetes Atlas 10th edition. IDF DIABETES ATLAS. 10th ed. Brussels: International Diabetes Federation; 2021.

[7] Pradeepa R, Mohan V (2021). Epidemiology of type 2 diabetes in India. Indian J Ophthalmol.

[8] Dixit AA, Azar KM, Gardner CD, Palaniappan LP (2011). Incorporation of whole, ancient grains into a modern Asian Indian diet to reduce the burden of chronic disease. Nutr Rev.

[9] Livesey G, Taylor R, Hulshof T, Howlett J (2008). Glycemic response and health--a systematic review and meta-analysis: relations between dietary glycemic properties and health outcomes. Am J Clin Nutr.

[10] Chawla R, Madhu SV, Makkar BM, Ghosh S, Saboo B, Kalra S (2020). RSSDI-ESI Clinical Practice recommendations for the management of type 2 diabetes mellitus 2020. Indian J Endocrinol Metab.

[11] Davies MJ, Aroda VR, Collins BS (2022). Management of hyperglycemia in type 2 diabetes, 2022. A consensus report by the American Diabetes Association (ADA) and the European Association for the Study of Diabetes (EASD). Diabetes Care.

[12] Garber AJ, Abrahamson MJ, Barzilay JI (2018). Consensus statement by the American Association of Clinical Endocrinologists and American College of Endocrinology on the comprehensive type 2 diabetes management algorithm - 2018 executive summary. Endocr Pract.

[13] Burroughs TE, Desikan R, Waterman BM, Gilin D, McGill J (2004). Development and validation of the diabetes quality of life brief clinical inventory. Diabetes Spectr.

[14] Reviriego J, Gomis R, Maranes JP, Ricart W, Hudson P, Sacristan JA (2008). Cost of severe hypoglycaemia in patients with type 1 diabetes in Spain and the cost-effectiveness of insulin lispro compared with regular human insulin in preventing severe hypoglycaemia. Int J Clin Pract.

[15] Carbohydrates in human nutrition (1998). Report of a Joint FAO/WHO Expert Consultation. FAO Food Nutr Pap.

[16] Zafar MI, Mills KE, Zheng J, Regmi A, Hu SQ, Gou L, Chen LL (2019). Low-glycemic index diets as an intervention for diabetes: a systematic review and meta-analysis. Am J Clin Nutr.

[17] Yu YT, Fu YH, Chen YH, Fang YW, Tsai MH (2025). Effect of dietary glycemic index on insulin resistance in adults without diabetes mellitus: a systematic review and meta-analysis. Front Nutr.

[18] Lattimer JM, Haub MD (2010). Effects of dietary fiber and its components on metabolic health. Nutrients.

[19] Brand-Miller JC, Holt SH, Pawlak DB, McMillan J (2002). Glycemic index and obesity. Am J Clin Nutr.

[20] Intensive blood-glucose control with sulphonylureas or insulin compared with conventional treatment and risk of complications in patients with type 2 diabetes (UKPDS 33). UK Prospective Diabetes Study (UKPDS) Group (1998). Lancet.

[21] Chiavaroli L, Lee D, Ahmed A (2021). Effect of low glycaemic index or load dietary patterns on glycaemic control and cardiometabolic risk factors in diabetes: systematic review and meta-analysis of randomised controlled trials. BMJ.

[22] Brand-Miller J, Hayne S, Petocz P, Colagiuri S (2003). Low-glycemic index diets in the management of diabetes: a meta-analysis of randomized controlled trials. Diabetes Care.

[23] Jenkins DJ, Kendall CW, Augustin LS (2002). Glycemic index: overview of implications in health and disease. Am J Clin Nutr.

[24] Guyatt GH, Feeny DH, Patrick DL (1993). Measuring health-related quality of life. Ann Intern Med.

[25] Gill TM, Feinstein AR (1994). A critical appraisal of the quality of quality-of-life measurements. JAMA.

[26] Tang TS, Funnell MM, Oh M (2012). Lasting effects of a 2-year diabetes self-management support intervention: outcomes at 1-year follow-up. Prev Chronic Dis.

[27] Augustin LS, Kendall CW, Jenkins DJ (2015). Glycemic index, glycemic load and glycemic response: an International Scientific Consensus Summit from the International Carbohydrate Quality Consortium (ICQC). Nutr Metab Cardiovasc Dis.

[28] Jenkins DJ, Kendall CW, McKeown-Eyssen G (2008). Effect of a low-glycemic index or a high-cereal fiber diet on type 2 diabetes: a randomized trial. JAMA.

[29] Thomas DE, Elliott EJ, Baur L (2007). Low glycaemic index or low glycaemic load diets for overweight and obesity. Cochrane Database Syst Rev.

